# Prevalence, influencing factors, and outcomes of emergency caesarean section in public hospitals situated in the urban state of Lagos, Nigeria

**DOI:** 10.4314/ahs.v23i2.74

**Published:** 2023-06

**Authors:** Aduragbemi Banke-Thomas, Cephas Ke-on Avoka, Olakunmi Ogunyemi

**Affiliations:** 1 School of Human Sciences, University of Greenwich, London, United Kingdom; 2 LSE Health, London School of Economics and Political Science, London, United Kingdom; 3 Faculty of Public Health, Ghana College of Physicians and Surgeons, Accra, Ghana; 4 Ministry of Health, Lagos State Government, Lagos, Nigeria

**Keywords:** Caesarean section, emergency obstetric care, prevalence, factors, urban, Nigeria

## Abstract

**Background:**

Caesarean section (CS) performed in an emergency can be life-saving for both the pregnant woman and her baby. In Nigeria, CS rates have been estimated to be 2.7% nationally, with the highest regional rate of 7.0% reported in the South-West of the country. Our objective in this facility-based retrospective cross-sectional study was to describe patterns and assess factors, obstetric indications, and outcomes of emergency CS in Lagos, Nigeria.

**Methods:**

Socio-demographic, travel, and obstetric data of pregnant women were extracted from case notes. Travel data was inputted in Google Maps to extract travel time from the pregnant women' home to the hospital. Univariate, bivariate and multivariable logistic regression analyses were conducted.

**Results:**

Of the 3,134 included pregnant women, 1,923 (61%) delivered via emergency CS. The odds of an emergency CS were significantly higher among women who were booked (OR=1.97, 95%CI 1.64–2.35), presented with obstructed labour (OR=2.59, 95%CI 1.68–3.99), pre-eclampsia/eclampsia (OR=1.67, 95%CI 1.08–2.56), multiple gestations (OR=2.71, 95%CI 1.72–4.28) and travelled from suburban areas (OR=1.43, 95%CI 1.15–1.78). There was an increasing dose-effect response between travel time to the hospital and emergency CS.

**Conclusion:**

Optimisation of CS rates requires a multi-pronged approach during pregnancy and childbirth, with particular emphasis on supporting pregnant women living in the suburbs.

## Introduction

Globally, 295,000 deaths occur annually as a result of complications of pregnancy and childbirth. These complications include bleeding, hypertension, infection, and abortion. Almost all these deaths occur in low- and middle-income countries (LMICs), with Nigeria alone accounting for over two-fifths of the global burden of maternal deaths^1^. A surgical intervention, known as Caesarean section (CS), is part of a package of clinical interventions known as emergency obstetric care (EmOC), which is routinely performed to prevent these deaths^2^. A CS is a form of foetal birth whereby the abdomen and uterus are surgically opened to extract the foetus. The surgery is performed either electively, with advanced scheduling, or as an emergency procedure and is expected to be carried out in hospitals classed as comprehensive EmOC facilities 2. The short-term benefit of CS, especially in an emergency, as opposed to an elective, is its life-saving function for both the pregnant woman and her baby. Evidenced long-term benefits include decreased risk of urinary incontinence and pelvic organ prolapse^3^. Contrarily, a CS also risks leading to a uterine rupture, abnormal placentation, ectopic pregnancy, stillbirth, and preterm birth for subsequent pregnancies 4.

While, for the most part, a medical practitioner makes the decision on the need for a CS, it is increasingly being performed at the request of pregnant women who have no medical indication for a CS. The global average CS rate has increased from 6.7% in the early '90s to 21.1% in the current decade. Though Africa has had the lowest rates in this period, there has been an almost three-fold increase in CS rates on the continent from 2.9% in 1990 to 7.4% in 2014 5,6. In Nigeria, where 40% of global maternal deaths occur 1, the national CS rate has been estimated to be 2.7%, as per the most recent published analysis of the 2018 Nigeria Demographic and Health Survey (NDHS) 7. Disaggregated by region, the South-West of the country has the highest regional CS rate at 7.0% based on the same 2018 DHS 7. These values are increments from the previous estimates of 2.1%nationally and 4.7% in the southwest reported in the 2013 NDHS 8.

Several facility-based studies on CS in Nigeria have been conducted in one hospital without capturing patterns across the health system 9–19. For those that explored associated factors for a CS, the focus has mostly been on socio-demographic and obstetric factors, with no attention paid to factors related to travel and accessibility to the service. Essentially, the focus in the literature thus far has addressed the first and third delay phases of the three-delay model, which relate to delay in the decision to seek care and delay to receive care upon arrival at the health facility. No previous research had explored the influences of the second delay phase, which relates to travel to reach care 20. This is despite evidence suggesting that in some cases, delays in arrival leave the skilled health personnel with no other option than an emergency CS 21. Furthermore, as per a quick review of the literature, almost all existing studies have looked at CS generally and not focused on emergency CS, which is particularly critical for survival. In this study, our objective was to describe patterns and assess factors, obstetric indications, and outcomes of emergency CS in Lagos State, Nigeria.

## Methods

Situated in the southwestern part of Nigeria, Lagos State is the economic nerve centre and the most urbanised state of the country. The state has diverse geographical terrains (including land and water) and settlement types (including its central megacity, suburbs, slums, and towns). Indeed, its centrally located megacity is the largest in sub-Saharan Africa. While predominantly urban, Lagos state has some rural parts in its extreme east and west. As per the most recent estimates in 2019, Lagos state has a population of approximately 26 million 22. The commonest mode of travel in the state is by road. However, in many areas of Lagos State, the road infrastructure is weak, which is underscored by the presence of several potholes that occasionally make roads inaccessible for travellers. Severe traffic jams are an everyday feature in the state, with flooding during the rainy season worsening road conditions. Repair works done to the roads are mostly patched up, which sometimes leads to even more travel disruptions 23–25.

We conducted a retrospective cross-sectional study of pregnant women with a gestational age of 28 weeks or more who presented with an obstetric emergency at any of the 24 public hospitals in Lagos between 1st November 2018 and 30th October 2019. Women delivered via CS or spontaneous vagina delivery (SVD) were included. However, we excluded women delivered via assisted vaginal birth (AVB) - about 4% of the initial sample. The public hospitals are all comprehensive EmOC facilities that provide CS routinely in the state 26. Generally, pregnant women in Lagos have reported a favourable opinion of the technical expertise in these facilities. However, the responsiveness of skilled health personnel working in these facilities remains a concern 27.

Reviewing clinical records of the included women, we extracted socio-demographic, travel, and obstetric data from patient case notes. Socio-demographic data collected included age, marital status, educational level attained, and employment status. To characterise travel, information on the address of patients, which is routinely collected as part of the registration of new cases, was collected. This location was used as the start point for journeys to care. Referral points, which are typically reported to various levels of completeness in notes during history taking, were collected. The destination was the hospital where the CS was performed. The travel data collected was exported to Google Maps, where typical travel time for the period of day they travelled was extracted. Using information from the clinical records showing the main obstetric complication that was managed, the member of the dyad (mother, foetus, or both) who had the obstetric complication for which the pregnant woman presented was identified. The definition of all variables is presented in Table 1.

**Table 1:** Definition of variables

Variables	Definition	Treatment
Emergency Caesarean section	Woman delivered via CS after presenting with an obstetric emergency	Binary variable taking the value of “1” if woman was delivered via CS, and “0” if woman delivered spontaneously.
**Independent**		
**variables**		
Age group (in years)	Age of the mother as indicated in the health records classified based on the obstetric risk profile	Categorical variable
Marital status	Marital status of the mother	Categorical variable
Educational level attained	Highest educational level of the mother	Categorical variable
Employment status	Employment status of the mother	Categorical variable
Parity	Number of births of the pregnant woman in her lifetime	Categorical variable
Number of gestations	Number of foetuses carried in the index pregnancy	Categorical variable
Booking status	Was the pregnant woman described as registered (booked) for antenatal care in the index pregnancy?	Yes or no
Maternal complication	Obstetric complication of the mother for which she was managed after presenting at the emergency	Categorical variable
Member of dyad with complication	Who had the complication for which the pregnant woman then presented in an emergency?	Categorical variable
Settlement type of place of residence	Type of settlement the woman lives: urban, suburban, or rural, as defined by the Lagos State Ministry of Land and Housing	Categorical variable
Weekend travel to facility	Did the pregnant woman travel to deliver in a health facility during the weekend?	Yes or no
Period of day of travel to facility	Period of the day pregnant woman presented at the hospital	Categorial variable
Referral	Referred from one health facility to one of the hospitals	Yes or no
Type of referral facility	Type of referral facility for those who were referred to the point of care including another hospital (public), another hospital (private), clinic, primary health centre, traditional birth attendant, nursing/maternity home, and non-formal referral centres like religious bodies	

Following univariate analysis of the outcome and independent variables to provide a descriptive overview of our study population, bivariate analysis was conducted to assess the strength of the association of independent factors on the mode of delivery. All plausible independent factors associated with birth and those with statistical significance (p<0.05) or those with a p-value greater than 0.05 but less than 0.10 were entered into a multivariable logistic regression to assess the effect of each variable independently on the mode of delivery while controlling for potential confounding effects of covariates. Adjusted odds ratios (OR) and the corresponding 95% confidence intervals (CI) were estimated. Missing data, including those of women whose journey from home to the hospital of care could not be determined, were managed by exclusion in all analyses. All statistical analyses were performed using Stata SE version 15.0 (StataCorp, College Station, Texas, USA).

Ethical approval for this study was obtained from the Research and Ethics Committees of the Lagos State University Teaching Hospital (LASUTH) (LREC/06/10/1226) and Lagos University Teaching Hospital (LUTH) (ADM/DCST/HREC/APP/2880). Social approval for the study was received from the Lagos State Government (LSHSC/2222/VOLII/107).

## Results

In all, 3,134 pregnant women who presented with an obstetric emergency were included in our sample, with 1,923 (61%) delivered via emergency CS. The proportion of births via emergency CS was higher amongst pregnant women who were 20 to 34 years old (75%), married (97%), self-employed petty traders (45%), multiparous (59%), with singleton pregnancies (94%), not registered for antenatal care at the hospital in which the birth took place (un-booked) (51%), presented with obstructed labour as a complication (38%) and with complications attributable to mother and foetus (65%). The proportion of births via emergency CS was also highest amongst those who travelled during the week (78%), morning (38%), from a suburban area (61%), and directly to a public hospital (72%) which was most likely a non-apex facility (81%). If they were referred, most (39%) came from primary health care centres. Most needed travel of 10-29 minutes to a public hospital (37%) (Table 2).

**Table 2:** Socio-demographic, obstetric and travel to care characteristics of pregnant women who presented with an obstetric emergency in Lagos, Nigeria

Characteristics	Total ([%] N=3,134)	CS ([%] n=1,923)	SVD ([%] n=1,211)
Age group			
12-19 years	76 (2.4)	41 (2.1)	35 (2.9)
20-34 years	2,322 (74.1)	1,432 (74.5)	890 (73.5)
35-50 years	736 (23.5)	450 (23.4)	286 (23.6)
Marital status			
Single	133 (4.2)	65 (3.4)	68 (5.6)
Married	3,001 (95.8)	1,858 (96.6)	1,143 (94.4)
Educational level attained (n=539)			
Primary	32 (5.9)	19 (4.9)	13 (8.5)
Secondary	280 (52.0)	188 (48.7)	92 (60.1)
Tertiary	227 (42.1)	179 (46.4)	48 (31.4)
Employment status			
Unemployed/Housewife	538 (17.2)	340 (17.7)	198 (16.4)
Student	159 (5.1)	79 (4.1)	80 (6.6)
Self-employed (Petty-trader)	1,416 (45.2)	855 (44.5)	561 (46.3)
Self-employed (Mid-high business)	345 (11.0)	225 (11.7)	120 (9.9)
Employed	676 (21.6)	424 (22.1)	252 (20.8)
Parity			
Nulliparous (0)	1,106 (35.3)	749 (38.9)	357 (29.5)
Multiparous (1-4)	1,953 (62.3)	1,136 (59.1)	817 (67.5)
Grand-multiparous (5 or more)	75 (2.4)	38 (2.0)	37 (3.1)
Number of gestation(s)			
Singleton	2,971 (94.8)	1,805 (93.9)	1,166 (96.3)
Multiple	163 (5.2)	118 (6.1)	45 (3.7)
Booking status			
Booked	1,357 (43.3)	941 (48.9)	416 (34.4)
Un-booked	1,777 (56.7)	982 (51.1)	795 (65.6)
Maternal complication			
No maternal complication	128 (4.1)	65 (3.4)	63 (5.2)
Obstructed labour	956 (30.5)	722 (37.6)	234 (19.3)
Haemorrhage	685 (21.9)	312 (16.2)	373 (30.8)
Pre-eclampsia/eclampsia	884 (28.2)	582 (30.3)	302 (24.9)
Sepsis	163 (5.2)	33 (1.7)	130 (10.7)
Others	318 (10.2)	209 (10.9)	109 (9.0)
Member of dyad with complication (n=3,087)			
Complications attributable to mother only	861 (27.9)	649 (34.3)	212 (17.8)
Complications attributable to foetus only	46 (1.5)	6 (0.3)	40 (3.3)
Complications attributable to mother and foetus	2,180 (70.6)	1,240 (65.4)	940 (78.9)
Settlement type of place of residence			
Urban	688 (21.9)	418 (21.7)	270 (22.3)
Suburban	1,730 (55.2)	1,163 (60.5)	567 (46.8)
Rural	716 (22.8)	342 (17.8)	374 (30.9)
Weekend travel to facility			
Yes	727 (23.2)	427 (22.2)	300 (24.8)
No	2,407 (76.8)	1,496 (77.8)	911 (75.2)
Period of day of travel to the facility (n=2,069)			
Morning	766 (37.0)	475 (38.1)	291 (35.4)
Afternoon	544 (26.3)	339 (27.2)	205 (25.0)
Evening	466 (22.5)	265 (21.2)	201 (24.5)
Night	293 (14.2)	169 (13.5)	124 (15.1)
Referral			
Not referred	2,267 (72.3)	1,383 (71.9)	884 (73.0)
Referred	867 (27.7)	540 (28.1)	327 (27.0)
Type of referral facility (N=867)			
Another hospital (public)	137 (15.8)	108 (20.0)	29 (8.8)
Another hospital (private	173 (19.9)	118 (21.9)	55 (16.8)
Clinic	64 (7.4)	42 (7.8)	22 (6.7)
Primary health centre	377 (43.5)	210 (39.0)	167 (50.9)
Traditional birth attendant	90 (10.4)	48 (8.9)	42 (12.8)
Nursing/maternity home	6 (0.7)	1 (0.2)	5 (1.5)
Non-formal referral	20 (2.3)	12 (2.2)	8 (2.4)
Type of facility of birth			
Apex facility	574 (18.3)	366 (19.0)	208 (17.2)
Non-apex facility	2,560 (81.7)	1,557 (81.0)	1,003 (82.8)

As per the bivariate analysis, marital status, employment, parity, number of gestations, booking status, maternal complication, member of a dyad with complication, settlement type of place of residence, referral, and type of referral facility were statistically significant factors (Table 3).

**Table 3:** Bivariate analysis exploring the proportions of emergency caesarean sections by socio-demographic, obstetric history, health system and travel to birth

Characteristics	Women delivered via emergency CS ([%]n=1,923)	Women delivered via SVD ([%] n=1,211)	*p*-value
Age group			
12-19 years	41 (53.9)	35 (46.1)	0.392
20-34 years	1,432 (61.7)	890 (38.3)	
35-60 years	450 (61.1)	286 (38.9)	
Marital status			
Single	65 (48.9)	68 (51.1)	0.003
Married	1,858 (61.9)	1,143 (38.1)	
Employment status			
Unemployed/Housewife	340 (63.2)	198 (36.8)	0.010
Student	79 (49.7)	80 (50.3)	
Self-employed (Petty-trader)	855 (60.4)	561 (39.6)	
Self-employed (Mid-high business)	225 (65.2)	120 (34.7)	
Employed	424 (62.7)	252 (37.3)	
Parity			
Nulliparous (0)	749 (67.7)	357 (32.3)	<0.001
Multiparous (1-4)	1,136 (58.2)	817 (41.8)	
Grand-multiparous (5 or more)	38 (50.7)	37 (49.3)	
Number of gestation(s)			
Singleton	1,805 (60.7)	1,166 (39.3)	0.003
Multiple	118 (72.4)	45 (27.6)	
Booking status			
Booked	941 (69.3)	416 (30.7)	<0.001
Un-booked	982 (55.3)	795 (44.7)	
Maternal complication			
No maternal complication	65 (50.8)	63 (49.2)	<0.001
Obstructed labour	722 (75.5)	234 (24.5)	
Haemorrhage	312 (45.6)	373 (54.4)	
Pre-eclampsia/eclampsia	582 (65.8)	302 (34.2)	
Sepsis	33 (20.3)	130 (79.7)	
Others	209 (65.7)	109 (34.3)	
Member of dyad with complication (n=3,087)			
Complications attributable to mother only	649 (75.4)	212 (24.6)	<0.001
Complications attributable to foetus only	6 (13.0)	40 (87.0)	
Complications attributable to mother and foetus	1,240 (56.9)	940 (43.1)	
Settlement type of place of residence			
Urban	418 (60.8)	270 (39.2)	<0.001
Suburban	1,163 (67.2)	567 (32.8)	
Rural	342 (47.8)	374 (52.2)	
Weekend travel to facility			
Yes	427 (58.7)	300 (41.3)	0.097
No	1,496 (62.2)	911 (37.8)	
Period of day of travel to the facility (n=2,069)			
Morning	475 (62.0)	291 (38.0)	0.172
Afternoon	339 (62.3)	205 (37.7)	
Evening	265 (56.9)	201 (43.1)	
Night	169 (57.7)	124 (42.3)	
Referral			
Not referred	1,383 (61.0)	884 (39.0)	0.511
Referred	540 (62.3)	327 (37.7)	
Referral facility (n=867)			
Another hospital (public)	108 (78.8)	29 (21.2)	<0.001
Another hospital (private	118 (68.2)	55 (31.8)	
Clinic	42 (65.6)	22 (34.4)	
Primary health centre	210 (55.7)	167 (44.3)	
Traditional birth attendant	48 (53.3)	42 (46.7)	
Nursing/maternity home	1 (16.7)	5 (83.3)	
Non-formal referral	12 (60.0)	8 (40.0)	
Total travel time (n=2,673^†^)			
0 – 9 minutes	284 (57.6)	209 (42.4)	0.219
10 – 29 minutes	622 (63.8)	353 (36.2)	
30 – 59 minutes	400 (63.0)	235 (37.0)	
60 – 119 minutes	282 (62.4)	170 (37.6)	
120 – 480 minutes	75 (63.6)	43 (36.4)	
Type of facility of birth			
Apex facility	366 (63.8)	208 (36.2)	0.114
Non-apex facility	1,557 (60.8)	1,003 (39.2)	

† Excludes women whose journey to the hospital could not be determined (n=461)

‡ Excludes women whose journey from home to the health facility could not be determined (n=116)

Following adjustments in multivariable analysis, the odds of an emergency CS were significantly higher among women who were married (OR=1.71, 95%CI 1.09–2.68), booked for antenatal care (OR=1.97, 95%CI 1.64–2.35), presented with obstructed labour (OR=2.59, 95%CI 1.68–3.99), pre-eclampsia/ eclampsia (OR=1.67, 95%CI 1.08–2.56), and other complications including retained placenta, malpresentation and malposition at term, and post-dated pregnancies (OR=1.70, 95%CI 1.05–2.75) as well as women with multiple gestations (OR=2.71, 95%CI 1.72–4.28). Odds were also significantly higher for pregnant women who needed to travel 10-29 minutes (OR=1.33, 95%CI 1.04–1.69), 30-59 minutes (OR=1.48, 95%CI 1.13–1.94), 60-119 minutes (OR=1.78, 95%CI 1.33–2.38), 120-480 minutes (OR=2.79, 95%CI 1.142.80), and from suburban areas (OR=1.43, 95%CI 1.15–1.78). However, the odds of an emergency CS were significantly lower among women who were students (OR=0.51, 95%CI 0.33–0.77), presented with sepsis (OR=0.24, 95%CI 0.13–0.43), and from rural settlements (OR=0.53, 95%CI 0.41–0.68) (Table 4).

**Table 4:** Multivariable logistic regression models for the odds of delivery by caesarean section

Factor	Unadjusted model (95% CI)	Adjusted model (95% CI)
Total travel time (N=2,673^†^)		
0 – 9 minutes	1.00	1.00
10 – 29 minutes	1.30 (1.04 – 1.62) *	1.33 (1.04 – 1.69) *
30 – 59 minutes	1.25 (0.98 – 1.59)	1.48 (1.13 – 1.94) **
60 – 119 minutes	1.22 (0.94 – 1.58)	1.78 (1.33 – 2.38) ***
120 – 480 minutes	1.28 (0.85 – 1.94)	2.24 (1.43 – 3.51) ***
Booking status		
Un-booked	1.00	1.00
Booked	1.83 (1.58 – 2.12) ***	1.97 (1.64 – 2.35) ***
Parity		
Nulliparous (0)	1.00	1.00
Multiparous (1-4)	0.66 (0.57 – 0.77) ***	0.67 (0.55 – 0.81) ***
Grand-multiparous (5 or more)	0.49 (0.31 – 0.78) **	0.55 (0.30 – 1.00)
Member of dyad with complication (n=3,087)		
Complications attributable to mother only	2.32 (1.94 – 2.77) ***	2.16 (1.76 – 2.64) ***
Complications attributable to foetus only	0.11 (0.05 – 0.27) ***	0.10 (0.04 – 0.26) ***
Complications attributable to mother and foetus	1.00	1.00
Maternal complications		
No maternal complication	1.00	1.00
Obstructed labour	2.99 (2.05 – 4.36) ***	2.59 (1.68 – 3.99) ***
Haemorrhage	0.81 (0.56 – 1.18)	0.79 (0.51 – 1.22)
Pre-eclampsia/eclampsia	1.87 (1.29 – 2.71) **	1.67 (1.08 – 2.56) *
Sepsis	0.25 (0.15 – 0.41) ***	0.24 (0.13 – 0.43) ***
Others	1.86 (1.23 – 2.82) **	1.70 (1.05 – 2.75) *
Settlement type of place of residence		
Urban	1.00	1.00
Suburban	1.32 (1.10 – 1.59) **	1.43 (1.15 – 1.78) **
Rural	0.59 (0.48 – 0.73) ***	0.56 (0.43 – 0.72) ***
Marital status		
Single	1.00	1.00
Married	1.70 (1.20 – 2.41) **	1.71 (1.09 – 2.68) *
Number of gestations		
Singleton	1.00	1.00
Multiple	1.69 (1.19 – 2.41) **	2.71 (1.72 – 4.28) ***
Employment status		
Unemployed/Housewife	1.00	1.00
Student	0.58 (0.40 – 0.82) **	0.51 (0.33 – 0.77) **
Self-employed (Petty-trader)	0.89 (0.72 – 1.09)	0.85 (0.66 – 1.08)
Self-employed (Mid-high business)	1.09 (0.82 – 1.45)	1.00 (0.72 – 1.38)
Employed	0.98 (0.77 – 1.24)	0.76 (0.58 – 1.00)
Age group		
12-19 years	0.73 (0.46 – 1.15)	1.01 (0.56 – 1.81)
20-34 years	1.00	1.00
35-60 years	0.98 (0.82 – 1.16)	1.04 (0.85 – 1.28)

Note: ***p<0.001; **p<0.010; *p<0.050; OR Odds Ratio, CI Confidence Interval

† Excludes women whose journey to the hospital could not be determined (n=501) Footnote (Model description): In the adjusted model, all variables have been adjusted for each other, including socio-demographic, health facility variables, and time of travel for delivery.

For outcomes, there were 18 (0.9%) maternal deaths following emergency CS compared to 30 (2.5%) after spontaneous birth. There were 155 (8.1%) stillbirths following emergency CS compared to 230 (19.0%) after spontaneous birth. These included 106 (5.5%) fresh stillbirths following emergency CS compared to 147 (12.1%) after spontaneous birth.

## Discussion

As per our study, emergency CS is commonly performed for pregnant women who present with obstetric emergencies in Lagos. This finding is not unexpected in this sub-population of pregnant women, being that they have presented with obstetric emergencies and required care in hospitals. A ten-year review of 12,811 deliveries in a Lagos tertiary public hospital showed that 51% of births were conducted via CS (elective and emergency) 11. The justification for performing an emergency CS in such critical situations is not particularly surprising, as the priority of skilled health personnel is to save the lives of the mother and the baby. However, it is still high considering that AVB is an alternative, which though we have not included them in our study, remain rarely performed (only about 4% done). Generally, AVBs have gradually declined in many LMICs, including Nigeria, and researchers have asked if the procedure still has a role 28. In our study, obstructed labour (38%), followed by pre-eclampsia/ eclampsia (30%) and haemorrhage (16%) were the commonest complications for which an emergency CS was conducted. Another study in Lagos showed that pre-eclampsia/eclampsia, haemorrhage, and foetal distress were the commonest complications 11. These complications contribute the most to maternal mortality 1; as such, it is totally reasonable that they are also the ones particularly necessitating an emergency CS.

In terms of factors associated with an emergency CS, we found that the odds for an emergency CS were significantly higher for those with obstructed labour, pre-eclampsia/eclampsia, retained placenta, malpresentation/malposition, multiple gestations, and post-dated pregnancies. Some of these have been reported previously in Lagos public hospitals 10,11. One finding that had not been previously reported is that being booked increased the odds of CS. A case-control study conducted in a teaching hospital based in Lagos highlighted that being un-booked increased the odds of CS 10. However, two studies that did not specifically test association reported high CS rates among booked patients 13,18. This finding might be related to some booked mothers deciding to deliver in other settings, not minding the birth plan that had been instituted at the booking facility but then returning to the facility after complications develop 13 or because the booked cases in referral hospitals, like those included in our study, are typically the highest risk cases anyways, and these tend to require a CS 18.

Additionally, we found that the odds of an emergency CS were significantly higher among married women. With elective CS, fear of pain and injury to mother and baby as a result of vaginal delivery and uncertainty regarding vaginal birth, and positive perception of CS have been given as reasons for women choosing CS 29,30. However, in our study that focuses on emergency CS, which would be expected to be offered to all women who need it, irrespective of their marital status, it is not clear why this is the case. Qualitative research with skilled health personnel might help to understand this association better.

Contrary to a previous cross-sectional study conducted in one of the Lagos teaching hospitals, which compared emergency CS and spontaneous vaginal births 9, we did not find maternal age a significant factor associated with emergency CS. However, our study findings align with this study regarding the association of multiple gestation, prolonged/obstructed labour, and haemorrhage. For the protective nature of being a student that we found in our study, despite multiple explorations, we are not able to explain this relationship, and it needs to be scrutinised further.

Another new finding that our study adds to the literature is the strong increasing dose-effect response for travel time to a public hospital. This finding was particularly intriguing because travel time was not significant in our bivariate analysis. This is not an unusual phenomenon, and it is likely due to some missing data with travel time estimation in our study since we could not trace where all the women came from to reach the hospital 31. However, as our research focused on women who presented with an obstetric emergency, this is a particularly important finding. Many pregnant women have significant challenges reaching a hospital when in an emergency 32. Our finding that longer travel time increases the odds of an emergency CS can be plausibly explained. Essentially, by the time women needing to travel for a more extended time to reach a public hospital, the skilled health personnel are left with minimal leeway to give her a trial of labour, as there is an urgency to save the mother and the unborn child 21. Alternatively, the strong association between increased time of travel and an emergency CS could be a measure of the inability of health facilities proximal to women with obstetric emergencies to provide this life-saving intervention, which causes the women to have to travel for a longer time to reach an appropriate facility.

We also found that the odds of an emergency CS were significantly higher for women travelling from suburban areas. For this relationship, the bad roads and traffic in the Lagos suburbs probably mean these women need to travel for a longer time or women living in the suburbs, who are mainly of the lower socio-economic status, travel to other facilities before being referred to a public hospital. It might also simply mean that women in the suburbs believe they are close by to a hospital and, as such, commence their journeys later when the only option for the skilled health personnel is to deliver them via a CS. Regarding the protective nature of rural residency that we observed in this setting, these women tend to travel to hospitals located within their communities, so they do not need to travel so far to access an emergency CS if needed 33.

In our study, maternal death following emergency CS remained <1%, lower than the approximately 2.5% observed amongst spontaneous births. This comparatively lower maternal death rate following emergency CS than spontaneous birth has been previously reported in Lagos 11. Our rates are also similar to estimates from another study conducted in Abuja, Nigeria 12. However, our rate across the state was lower than estimates of 6% from hospitals in other Nigerian regions 13. We also showed that rates of fresh stillbirths amongst women delivered spontaneously were more than twice the rates amongst those delivered via emergency CS.

A key strength of our study is that we have explored factors influencing emergency CS across all public hospitals within a health system of a Nigerian state. In addition, by focusing only on women with obstetric emergencies, we eliminate the effect of those who would have had a CS as elective surgery or out of personal choice. Furthermore, we have included theoretically driven independent variables in constructing our model, as per recommended best practice 34. In accounting for travel time, we have incorporated estimates using Google Maps, which have been shown to be closer to reality 35, compared to the other approaches used for accessing geographical accessibility in the literature 36. Limitations to bear in mind are that being a study based on existing records, our analysis is only as good as the details recorded. For example, we have assumed that all women travelled from their places of residence, but this might not be the case. We have also classified women as booked or un-booked based on how the woman was described in her clinical records at the public facility, but we do not know if she was registered elsewhere, for example, in a private clinic. Definition of booking status is a recognised challenge for facility-based studies 37. However, we did check with attending physicians when there were confusing notes recorded for the pregnant women. In addition, our travel time estimation does not include time for waiting for the car or waiting to receive care at the facility upon arrival. Furthermore, we have not included mode of transport, such as a private car or ambulance, which is critical for access to the service. This information is not typically reported in clinical records of pregnant women in these settings 37.

There are clear implications for practice and policy emanating from our findings. For clinical practice, though rates remain lower than those seen in many parts of the globe, CS rates remain high in Lagos public hospitals. Bearing in mind the established negative long-term effects associated with CS in general 4, skilled health personnel need to be equipped to effectively conduct assisted vaginal birth, especially for some obstetric complications that have higher odds of emergency CS. Skilled health personnel should also recognise that being booked does not guarantee reduced odds for emergency CS for women with an obstetric emergency, which cannot always be predicted. There is also a case for preparing the theatre for a potential emergency CS ahead of a pregnant woman's arrival, especially when the complication she is experiencing relates to her, as opposed to her unborn baby. For policy, though the government has suggested that public hospitals are strategically located across the state 26, there remains a critical need to support pregnant women with obstetric complications to reach hospitals in a quicker time, especially those living in the suburbs. To minimise unnecessary CS, efforts to ensure that skilled health personnel are well trained in recognising when a CS is particularly necessitated vs alternative birthing procedures can be safely explored. When CS is provided, this should be done in the most cost-effective manner 38,39.

Put together, our study adds to the body of evidence on emergency CS rates, patterns, and outcomes in an urban LMIC setting. In addition, it highlights an association between travel time and emergency CS in such settings. While CS rates still need to be optimised in general, our findings highlight the need not to neglect urban areas in such efforts, under the assumption that these settings are close to the city. Indeed, CS remains a life-saving intervention preventing many maternal and perinatal deaths.

In addition to addressing barrier issues such as cost of care 40, efforts to optimise CS will require the engagement of women all through pregnancy, supporting those with obstetric complications trying to reach a hospital and exploring other birthing alternatives before choosing a CS.
